# The 1984 Rajneeshee Bioterrorism Attack: An Example of Biological Warfare by Violent Non-state Actors

**DOI:** 10.7759/cureus.88514

**Published:** 2025-07-22

**Authors:** Matthew D Turner, Kimberly Marinconz, Griffin Shimp

**Affiliations:** 1 Emergency Medicine, Penn State Health Milton S. Hershey Medical Center, Hershey, USA

**Keywords:** biological warfare, bioterrorism, rajneeshee cult, salad bar, salmonella, terrorism, the dalles

## Abstract

With the advancement of technology and the life sciences, bioterrorism poses a unique and ever-evolving challenge to public security. In this article, we discuss one of the largest incidents of bioterrorism in the history of the United States. This attack highlights the unique threat that even resource-limited, small-scale bioterrorism poses to wider society when in the hands of small and highly motivated organizations. Physicians, scientists, and legislators should be well aware of the potential risk this poses in the coming years.

## Introduction and background

Bioterrorism is defined as an intentional release of a biological agent, as part of a covert or overt action, against a civilian population, for political, religious, or other ideological motivations [1]. Examples include the 1972 R.I.S.E. attempt to contaminate the water treatment systems of Chicago, the 2001 anthrax letters that were mailed to various companies and individuals across the United States [1], and a 2010 attempt by the Animal Liberation Front to send AIDS-contaminated razor blades through the mail [2]. However, the largest episode of bioterrorism in the United States was the Rajneeshee cult’s attempts to poison multiple restaurants in Oregon in 1984 with *Salmonella*, sickening over 750 people [1].

## Review

*Salmonella *species

*Salmonella *is a gram-negative bacterium with over 2500 recognized serotypes, and one of the most common causes of gastrointestinal disorders in the world [3]. While the prognosis is typically benign and self-limited, children, the elderly, and immunocompromised patients are especially vulnerable to this infection [3]. Non-typhoidal *Salmonella *infections typically present as a self-limiting illness and are characterized by nausea, vomiting, severe abdominal cramps, fever, and bloody diarrhea [3]. Food poisoning outbreaks are common, often from contaminated produce, raw eggs, seafood [3], meat products, and water [4]. Proper food hygiene is essential to the prevention of *Salmonella *infections [3]. The United States Centers for Disease Control and Prevention (CDC) ranks *Salmonella *species within Category B of their ranking of potential biological weapons. While this indicates that *Salmonella *species are less easily transmissible and have a lower morbidity and mortality compared to the agents within Category A, such as *Bacillus anthracis* and *Franciscella tularensis*, they still pose a significant threat as potential biowarfare agents [5].

A 2005 report by the *Bulletin of the Atomic Scientists *noted the potential for *Salmonella *as a bioterrorism weapon. It is common worldwide, has the potential to lead to serious complications, can be easily spread through the contamination of food and water supplies, and there is no vaccine available [6]. While it has less potential for mass morbidity and mortality than other agents, the unique ease of access means that *Salmonella *is uniquely poised as a small-scale weapon of bioterrorism [6].

History of *Salmonella *as a weapon

Germany

Over the past century, multiple nations have investigated *Salmonella *as a potential agent of biological warfare. Germany developed the world’s first documented biological warfare program during the First World War, attempting to spread multiple pathogens, including anthrax, in countries that included the United States, Russia, Spain, Norway, and France [1]. The German program continued even after the conclusion of hostilities; several of the bacterial agents they later investigated included *Salmonella* for the possible contamination of enemy water sources [1].

Interwar France

Inspired by Germany’s experiments with biological warfare (BW) during the First World War, the French government began to investigate the potential applications of widespread BW in the 1920s. The Trillat Report of 1922 argued for the dissemination of biological agents via aerial dispersion in a “microbial ‘cloud’ of fine droplets”. By October 1926, the program had developed a means of delivery system via aerial bombs that produced “clouds consisting of very small droplets containing fragmented microorganisms with the capability, despite their fragmentation, of producing pathogenic effects.” *Salmonella *was one of the organisms successfully tested in open-air experiments upon animal subjects. Experiments continued intermittently after the signing of the Geneva Protocol, in part spurred on by allegations that the Germans were conducting BW experiments of their own. By 1940, the Prophylaxis Commission also recommended the contamination of food, water, and medical supplies with biological agents such as typhoid. Despite experimentation and planning, ultimately the French surrender in 1940 precluded any deployment of biological warfare, and the program was suspended [7].

Imperial Japan

Imperial Japan had the largest and most sophisticated BW program of the Second World War. Under the supervision of Dr. Ishii Shiro, the program employed over 10,000 employees and had multiple facilities, including one in Manchuria that contained a private airstrip and over 150 buildings. Known as Unit 731, the program employed human experimentation on thousands of prisoners and produced massive quantities of pathogens employed in multiple types of biological bombs [7].

The full extent of the crimes of Unit 731 is outside the scope of this paper [8]; however, the widespread use of *Salmonella *species in field testing was an area of interest for the program. In 1939, during clashes between Japanese and Soviet forces along the Manchurian border, the unit delivered 2000 biological shells containing *Salmonella *to the front lines. It remains unclear how much these shells contributed to casualties during the battle; although many soldiers died of disease, much of that may have simply been due to the poor conditions in the area [7]. For the next 3 years, Unit 731 conducted extensive field testing throughout China, disseminating plague-infected rats and foodstuffs, as well as deliberately infecting wells and water reservoirs with *Salmonella *and other infectious agents [7]. Up to 1,000 Chinese wells were poisoned with cholera and *Salmonella Typhi *[9]. The true extent of casualties is impossible to determine, but it may have ranged from tens of thousands [10] to as many as 400,000 casualties [11].

Despite the atrocities inflicted by Unit 731, ultimately, its leadership was disappointed by the results. In spite of developing multiple different bombs designed to deliver biological agents, the program was unable to develop a “satisfactory” delivery system effective in warfare. By 1942, the program transitioned from field testing to human experimentation in a laboratory setting [7].

Israel

During the 1948 Israeli War of Independence, scattered reports from the International Red Cross led to accusations that the Haganah militia was deliberately poisoning the wells of the city of Acre with *Salmonella Typhi *bacteria, leading to a local typhoid outbreak [11]. Operation Cast Thy Bread remains a controversial topic which Israel has vociferously denied [12].

In 1999 and 2000, eggs sold from Israel were reportedly deliberately infected with *Salmonella*, prompting a recall. This appears to have been part of a campaign of economic disruption targeted against the Israeli food supply [13].

The 1984 Rajneeshee bioterrorism attack

The most infamous use of *Salmonella *as an agent of biological warfare occurred in Oregon in 1984 [14].

The Rajneeshees

From 1981 to 1985, followers of the spiritual leader Bhagwan Shree Rajneesh attempted to form an international commune in the isolated region of northern Oregon [15]. The Bhagwan, as he was often called, moved his community of followers from India to the 64,000-acre Big Muddy Ranch in June 1981. Under the supervision of his secretary, Ma Anand Sheela, the cult rapidly constructed a planned community called Rajneeshpuram. Rajneeshpuram had a permanent population of approximately 2,500 that rapidly swelled to as much as 15,000 during festivals. The cult’s facilities included a hospital, shopping mall, water and sewage services, and a regular police force [15].

The rapid expansion and development of Rajneeshpuram quickly led to tensions with local Oregonians [15]. One of the earlier flash points between the Rajneeshees and the surrounding community was over the land use of the former ranch, initially zoned as agricultural land, but which the cult wanted to establish a town on [16]. Within several months, the cult purchased multiple properties within the small nearby town of Antelope. As the cult’s influence grew, concerned locals attempted to disincorporate the town to prevent a Rajneeshee takeover. Taking advantage of Oregon’s voter registration laws, the Rajneeshees moved a large group into Antelope and won control of the Antelope City Council in 1982. Under the tight control of the cult, Antelope was renamed 'Rajneesh' and remade in the Bhagwan’s image. Streets were renamed, taxes raised to force out older residents relying on fixed incomes, an intimidating “Peace Force” patrolled the tiny town with semi-automatic rifles, and a new landfill was insultingly placed next to the local church and named the “Adolph Hitler Garbage Dump” [15]. Hostility between Oregonians and Rajneeshees soon escalated, with local protests and Rajneeshee counter-demonstrations soon becoming a regular occurrence throughout nearby communities [16].

Despite early successes in incorporating Rajneeshpuram as an official city within Oregon, the cult was stymied when the Oregon Supreme Court canceled the incorporation, ruling that the town violated the constitutional separation of church and state [16]. The ongoing conflict between the cult and the locals also began to threaten to turn violent, when in August 1983, a Rajneeshee-owned hotel in Portland was bombed [16]. In response, the Rajneeshees became more paranoid and militaristic; the cult purchased automatic weapons and began to have helicopter teams perform regular reconnaissance flights in the skies above Rajneeshpuram [15]. Security became more elaborate, with tight restrictions placed on travel and communication with the outside world [16]. Widespread wiretapping around the commune became extremely prevalent [16].

Powerful defenses were not enough; the Bhagwan determined that the community’s best chance for survival lay in aggressive expansion. After the cult took control of Antelope, it turned its attention to Wasco County’s leadership, a three-member governing board [15]. In the fall 1984 elections, the Rajneeshees planned to win at least two of the three judgeship positions of the Wasco County leadership [17], a move that would bring the entire county under the cult’s sphere of influence [15]. Heavily outnumbered by the native Oregonians, the cult resorted to underhanded tactics to rig the election in their favor [17]. Over 1500 Rajneeshees and sympathizers to the cult were encouraged to move to Rajneeshpuram over the preceding months and register to vote. Still outnumbered by the native population, the cult then unveiled its Share-a-Home program. In a bold move, the cult bused in over 3500 homeless people from across the United States and attempted to register them as Wasco County voters [15]. Even if this endeavor had succeeded, the cult would still have only garnered approximately 5,500 votes, compared to the 14,000 native Oregonian votes [17]. As it was, the county clerk, Sue Proffitt, halted voter registration on October 10 to prevent these thousands of newcomers from being able to vote [17]. The Rajneeshees quickly abandoned the Share-A-Home program and began to forcibly evict thousands of the homeless they had adopted into nearby towns [15]. However, the Rajneeshees had one more trick up their sleeve to secure the election.

1984 Bioterrorism Attack

It was in late summer 1984, during a brainstorming session about the upcoming election, that Ma Anand Sheela wondered, “What if people had the shits on Election Day?” [14]. The Rajneeshees were highly outnumbered by the Oregon electorate, especially in The Dalles, a nearby city with a population of approximately 10,500 [18] that had the highest number of voters in the region. If those voters could be kept away during the election, Sheela reasoned, then the Rajneeshees would have a chance [14]. She quickly became fascinated by the idea and turned to Ma Anand Puja for results [14].

Within Rajneeshpuram, the small Rajneesh Medical Corporation was run under the auspices of Ma Anand Puja, a licensed nurse originally from California. The corporation had expanded to meet the needs of the growing community, and eventually included two clinics, a laboratory, a pharmacy, and several offices [14]. Puja was devoted to Sheela and served as something of a “handmaiden” to her, with multiple other cult members theorizing that Puja was placed as head of the corporation due to her loyalty rather than to any innate ability [14]. This fit with the overall culture of leadership in the commune, where social life was tightly controlled and any sort of negative feedback from followers to leaders was strongly discouraged [16]. As part of the ‘siege mentality’ that had gripped the cult by late 1984, an atmosphere of paranoia and secrecy pervaded the commune, allowing leadership figures to become more forceful and tyrannical in their orders [16], further explaining Puja’s fanatical loyalty to Sheela [14].

By this point, Puja was no stranger to poisoning political enemies. Earlier in summer 1984, as factions within the commune wrestled for control, the Bhagwan’s personal physician had arrived at her clinic, complaining of diarrhea. While the physician was receiving IV fluids, Puja clumsily attempted to poison him by crudely injecting dirty water from a nearby vase of flowers into his IV drip. The physician soon went into sepsis and only barely survived; a blood culture grew significant amounts of *Citrobacter *in his blood [14]. However, Sheela and Puja were satisfied with the results, as the physician’s prolonged recovery sidelined him from the inner political workings of the cult for several weeks [14].

However, Sheela’s request for a mass poisoning of voters in The Dalles was on an entirely difficult scale. Under an atmosphere of total secrecy, Puja turned the Rajneesh Medical Corporation’s resources toward producing and disseminating a proper biological agent. Her control over the corporation was so ironclad and so secretive that most of her employees never had any idea of the project [14].

The resources dedicated to the project were limited and legally obtained, with only a small number of the cult’s members aware of the conspiracy, making for a tiny footprint [14]. Sheela requisitioned a small slat shed, colloquially known as the “Chinese Laundry”, that had been present before the Rajneeshees arrived, and Puja quickly converted it into a secretive laboratory with petri dishes, a freeze dryer, an incubator, and other equipment [14]. Hepatitis [14], AIDS, and *Salmonella Typhi *were among the biological agents considered [17], but eventually Puja decided upon *Salmonella typhimurium*. The Rajneesh Medical Corporation purchased samples of the pathogen from a medical company called VWR Scientific in Seattle; soon the “…Chinese Laundry became a salmonella factory operating at full steam…” [14]. The *Salmonella *was grown on Bactrol discs that were also legally purchased [1].

The group initially tested their *Salmonella *by contaminating glasses of water given to local officials visiting the ranch on August 29 [17]. Wasco County Judge Bill Hulse became so sick with severe stomach cramps the next day that he had to be hospitalized for four days and was reportedly on the verge of death [14]. That same month, Krishna Deva, the mayor of Rajneeshpuram, used an eyedropper to deliberately contaminate the doorknobs and urinals of government buildings [17], including the Wasco County courthouse [14]. Other early attempts included Deva pouring a vial of *Salmonella *into a bucket of salad dressing at the Portage Inn in The Dalles, as well as Puja and Sheela personally sprinkling samples of *Salmonella *over the produce section at a local grocery store [14]. “Let’s have some fun,” Sheela reportedly told Puja beforehand [14].

These early experiments did not yield the large-scale results that the Rajneeshees wanted [17]. However, the poisoning of the local officials did indicate that the *Salmonella *they had produced was effective. Sheela determined that it was time for the next step. In early September 1984, she assembled several teams and handed them vials of brown, foul-smelling liquid. “If there is a choice between one thousand unenlightened people or one enlightened master, you should always choose the enlightened master,” she told one of her assistants [14]. The chosen crews dressed in nondescript clothing and infiltrated 10 restaurants across The Dalles, sprinkling their *Salmonella *samples across salad bars, slipping it into coffee creamer, and outright pouring it into salad dressing. Puja personally oversaw much of the operation and was said to have “poured the salmonella with such panache” that other members of the cult were concerned that she would be discovered [14].

The first cases of food poisoning were reported on September 9, 1984 [14] and were identified as *Salmonella typhimurium* by local physicians within 4 days [17]. By September 17, the Wasco-Sherman Public Health Department began to investigate an increasingly large outbreak of severe gastroenteritis across The Dalles [18]. Many of the patients reported eating food from local salad bars, and so on September 25, authorities shut down all salad bars in the town. Due to the sheer scale, the Centers for Disease Control (CDC) intervened to assist the Oregon Health Division in investigating the outbreak [18].

While there were no fatalities from the outbreak, the scale of it was staggering. Multiple patients had to be hospitalized, including a 34-year-old pregnant woman who gave birth to a neonate in septic shock from *Salmonella *poisoning [14]. Both the mother and the infant barely survived the ordeal [14]. In total, 751 patients experienced severe gastrointestinal poisoning in The Dalles [17]. Investigators found that the outbreak was divided into two distinct waves - one that ranged from September 9 to September 18 [18], affecting at least 88 people [14], and a second outbreak from September 19 to October 10 [18] that affected at least 586 people [14]. Sue Proffitt, the county clerk who had denied the Rajneeshee attempt to register thousands of new voters earlier, was among those poisoned [17]. Most of the infections could be traced back to 10 of the 36 restaurants located in The Dalles [18]. Several unusual aspects of the *Salmonella *involved in the outbreak soon became apparent - investigators found that it did not ferment dulcitol, a characteristic present in only 2% of nontyphoidal *Salmonella *[18]. All of the outbreaks in each restaurant had an identical plasmid profile, suggesting a single origin for the disease [18]. However, the source of infection was significantly different in each restaurant, ranging from blue cheese salad dressing (but not the dry mix it was originally prepared from) [18] to lettuce and potato salad in other restaurants [14]. Given this, investigators concluded in November 1984 that the outbreak was due to improper food handling. However, this was far from a satisfactory answer - food prepared in the kitchen did not appear to be a source of infection, nor were customers who ordered takeout infected by the *Salmonella *[14]. Most concerning of all, individual banquets within the affected restaurants were also unaffected, despite using the same food, with the same food handlers [19]. With at least 10 different restaurants affected, the conclusion that the outbreak was simply due to improper food handling practices seemed tenuous at best [17].

Despite the highly unusual circumstances of the outbreak, no suspicion fell on the Rajneeshees for months [14]. It wasn’t until Congressman James Weaver, one of the Oregon representatives to the House of Representatives, made a speech entitled “The Town that was Poisoned” to Congress on February 28, 1985 [19] that the cult was publicly associated with the poisonings [14]. Congressman Weaver concluded in his speech that “… the circumstances are so overwhelming in pointing to such deliberate contamination that … I can only conclude … that sabotage did take place” [19]. He pointed out that the Rajneesh Medical Corporation had the facilities and the resources to mass-produce *Salmonella*, that Ma Anand Sheela had often been quoted as saying, “Wasco County is so bigoted it deserves to be taken over,” and even discussed the suspicious poisonings of the officials that had visited Rajneeshpuram on August 29 [19]. He ended his speech by calling for a criminal investigation of the cult [19]. Weaver was widely disparaged and accused of being paranoid and prejudiced against the Rajneeshees [20].

Despite the scale of the outbreak, the Rajneeshees soon lost interest in further acts of large-scale bioterrorism, in part due to increased scrutiny from public health officials, the difficulty of replicating the feat, and the failure of the Share-A-Home program [14]. The cult’s loss in the 1984 elections further worsened the situation [14].

The ultimate collapse of Rajneeshpuram and the cult of Bhagwan is outside the scope of this paper, but within a year, the cult had nearly completely collapsed due to legal difficulties and multiple criminal investigations [14]. During the authority’s investigations, multiple members of the cult confessed to the mass *Salmonella *poisonings that had affected The Dalles. Investigators from Oregon state and the Federal Bureau of Investigation ultimately discovered the cult’s laboratories, as well as the open vial of *Salmonella typhimurium* originally used to mass-produce the *Salmonella*. It was identical to the outbreak strain in its antibiogram, biochemical markers, and its plasmid profile [18]. In April 1986, Sheela pleaded guilty to the charges of poisoning, along with multiple other criminal charges [14]. She ultimately only served 3 years in prison, after which she had her citizenship revoked and was banned from the United States for the rest of her life [14]. After being banned from the United States, the charismatic Bhagwan died just a few years later at the age of fifty-eight [14].

Small-scale bioterrorism

Bioterrorism attacks are extremely rare; one 2022 study estimated that they only comprise 0.02% of all historic terrorist attacks [21]. In the vast majority of cases, terrorist organizations, also known as Violent Non-State Actors (VNSAs), appear to strongly prefer the use of conventional weapons [22]. Even when biological agents are used, they typically result in fewer casualties than conventional weapons [23]. One reason for this is that large-scale attacks with biological agents are very difficult to execute, even for well-funded and organized groups. The apocalyptic cult Aum Shinrikyo, responsible for the infamous Tokyo subway attack, possessed “considerable wealth and scientific expertise”, but still failed in 10 separate attempts to employ aerosolized anthrax and botulinum toxins in an urban setting [24]. Still other obstacles include obtaining the proper biological agent - as noted in a 2001 *Bulletin of the Atomic Scientists *article, different strains of rinderpest have wildly different rates of lethality and ease of transmission. Different pathogens may require specialized equipment to culture and develop, especially depending on the routes of infection intended. Even assuming a pathogen is properly acquired and developed, most pathogens are sensitive to environmental conditions and will require further processing before the difficult process of dissemination [13]. Aerosolized agents are notoriously difficult to effectively spread and are highly reliant upon environmental conditions, including wind and rain. The Aum Shinrikyo cult, arguably the most well-funded and technically advanced VNSA biological warfare program in history [23], found dissemination of its agents to be extraordinarily difficult. The cult attempted dispersing agents via a remote-controlled helicopter as a primitive drone in 1994 [22], driving a car with a specialized exhaust pipe to disseminate botulinum toxin in 1990 and 1993, and spreading massive plumes of anthrax bacteria from an eight-story building in 1993 [23]. In each of these cases, the cult was foiled by improperly prepared agents, adverse weather conditions, and other factors [23]. Even securing a proper agent was extremely difficult for the wealthy cult - failed attempts included an expedition to Zaire in 1992 to secure a sample of Ebola virus during an outbreak there [23].

Likewise, proper storage of biological agents is often difficult and requires specialized care, making them difficult to maintain in a form ready for dispersal [11]. As one example, anthrax is notoriously difficult to store for extended periods [11]. Given this, the possibility of a bioterrorism attack on the level of a Weapon of Mass Destruction (WMD) is extremely low [6]. Even when production is achieved, widespread dissemination of a biological weapon remains extremely difficult [25]. Despite its lethality, the sheer amount of ricin that would be required to create an effective aerosolized concentration for a mass-casualty scenario makes it extremely impractical for anything significantly larger than individual poisonings [26]. Other limitations include political considerations - some VNSAs may refrain from biological agents due to the fear of government crackdowns, alienation of potential supporters, or due to specific aims in a territory that preclude the use of bioterrorism [23]. Even actors that have the capabilities for widespread dissemination of biological warfare, such as nation-states, are unlikely to do so, both due to the fear of repercussions as well as the negligible battlefield utility that biological weapons possess [25].

In this respect, the Rajneeshee *Salmonella *attack of 1984 is a rare exception - especially since cults, prone to “paranoid, fantasy-prone” thinking are considered to “… be the least suited to meet the complex requirements for a BW [Biological Weapons] programme …” [23]. However, it is important to emphasize that the cult considered the mass poisoning only a test run and was disappointed with the results. “Puja, you’ve done a good job of making everyone sick,” one of the cult members later recalled Sheela telling one of her accomplices. “Too bad you didn’t make more people sick.” [14]. Over the next several months, the cult attempted multiple times to poison the water supply to The Dalles - attempts included pouring supplies of *Salmonella *directly into the water tanks, a scheme to release bacteria-laden beavers into the water, and even discussion of pouring a solution “blended-up” beaver through the iron bars that protected the water tanks - with no significant success. Given the chlorine treatments and purification supplies the city’s water supply went through, as well as the vast amounts of *Salmonella *that would have been required, it is extremely doubtful the cult had any chance at success [14]. Replicating the salad bar poisonings of September 1984 would have also been extremely difficult, given the increased scrutiny from public health officials [14].

However, while the threat of large-scale bioterrorism attacks is rare, the potential for smaller-scale use of cheap biological weapons remains. Given the low costs of obtaining and developing pathogens such as *Salmonella*, the possibility for “low intensity use of biological weapons” should still be regarded as a viable threat [27]. It is important to emphasize that the Rajneeshees obtained all of their materials legally. In 1984, the Rajneesh Medical Corporation purchased a sample of *Salmonella typhimurium* from the Seattle company VWR Scientific. In a simple shed, under the supervision of Ma Anand Puja, the cult mass-produced the *Salmonella *using simple petri dishes, an incubator, and a freeze-drier [14]. The entire effort had an extremely small footprint and was so secretive that most of the other members of the Rajneesh Medical Corporation had no idea that the project even existed [14]. Producing large quantities of bacteria is cheap and can be easily done with even rudimentary equipment and skills [18]. With continuing advances in research and development, biological weapons are likely to come within the capabilities of an increasing number of VNSAs, with production of dangerous material becoming easier and easier [25]. Even on an international level, nation-states are more likely to use biological warfare in small-scale, covert operations where plausible deniability is essential, such as the Bulgarian government’s 1978 ricin poisoning of dissident Georgii Markov [25]. In a more recent example, Professor Vitaly Melnikov, director of the Russian Federation’s Department of Rocket and Space Systems, died in August 2023 from “mushroom poisoning”. His death came several weeks after the first Russian lunar mission in 47 years failed under his supervision; he was allegedly punished for the national embarrassment [28]. This illustrates one of the greatest strengths of biological weapons: the plausible deniability associated with them. Even when there is a preponderance of evidence regarding their use, such as 10 restaurants simultaneously poisoned by *Salmonella*, the dominant paradigm often disregards even considering that intentional bioterrorism could be a cause [20].

Even the Rajneeshees, after their 1984 mass poisonings in The Dalles, soon switched back to targeted acts of bioterrorism against specific targets. As part of a last-ditch attempt to influence the 1984 campaign, one Rajneeshee woman named Yogini smeared *Salmonella *across her palms and then shook the hands at a political event in an attempt to poison rival politicians [14]. While the attempt did not work [14], it is more consistent with the small-scale use of bioterrorism that is much more commonly employed by various actors [25]. Other failed attempts included poisoning Dan Durow, a Wasco County official, with a thermos of contaminated coffee in early 1985 [14].

Recent years have demonstrated multiple other examples of bad actors using commercially available agents, intended for research purposes, as rudimentary CBW (chemical and biological weapons) agents. In 2023, a PhD student at the University of South Florida used samples of methadone and hydrocodone from the university’s lab in an attempt to poison his upstairs neighbors. On at least three separate occasions, he would inject a syringe of the two chemicals beneath his neighbors’ front door, aerosolizing the chemicals and causing significant health issues for the family and their newborn daughter [29]. In a similar vein, a laboratory technician at the American Tissue Culture Collection attempted to obtain a sample of the plague bacterium for purposes of political terrorism; he was only caught when he complained to colleagues that the procedure was taking too long [9]. Another lab worker in 1996 was arrested after deliberately poisoning coworkers with *Shigella dysenteriae* [30]. The insider threat from biological agents - in laboratory workers, researchers, and other staff - should not be underestimated [23]. As the U.S. Commission on the Prevention of Weapons of Mass Destruction, Proliferation and Terrorism phrased it, the world “… should be less concerned that terrorists will become biologists and far more concerned that biologists will become terrorists.” [20].

Other bad actors have attempted to purchase biological agents commercially. The anti-government group, the Minnesota Patriots Council, purchased castor beans through the mail in 1991 and successfully manufactured a small amount of ricin that they plotted to assassinate government officials with [31], via dermal absorption through doorknobs and clothing [32]. Several years later, an individual in Ohio was arrested after attempting to purchase cultures of bubonic plague through the mail [11]. In 2008, a man accidentally poisoned himself in Las Vegas while manufacturing ricin in a hotel room [26]. The increasing commercialization of biotechnology, such as Clustered Regularly Interspaced Short Palindromic Repeats (CRISPR) kits, only makes the potential for small-scale production of biological agents even greater in the coming years [22]. 

The COVID-19 pandemic has also increased VNSA interest in the use of bioterrorism; throughout the pandemic, multiple organizations such as the Council of Europe’s Committee on Counter-Terrorism warned that neo-Nazi groups were discussing the possible use of coronavirus as a biological weapon [22]. A number of manuals discussing the production of chemical and biological weapons currently circulate in radical spaces online, many of which detail the production of toxins such as ricin [22]. Other manuals detailing bioterrorism have been found in the hideouts of various VNSAs [23]. One instructional online video was used by a man in Cologne to produce significant amounts of ricin in 2018 [22], which he planned to combine with a crude homemade bomb for maximum dispersal. Fortunately, the perpetrator was arrested before he could disseminate the toxin [33]. Even literature such as the 1978 white supremacist novel *The Turner Diaries* can serve as inspiration for violent attacks - by Europol’s estimate, the book has inspired attacks resulting in over 200 deaths; many of the radicals inspired by the book were found to be stockpiling biological weapons [22]. While many of these online manuals are “amateurish” and often feature “directly incorrect information”, indicating a low level of sophistication, the fact that such manuals are circulating in increasing amounts online is indicative of a concerning increase in bioterrorism by VNSAs [22]. Even small-scale attacks have the potential to result in significant repercussions to wider society [23].

Given the threat posed by such “low-effort” biological agents [34], these examples demonstrate the importance of closely monitoring commercially available laboratory equipment and agents [35], as well as monitoring access to public laboratories [29]. In particular, the relative ease with which scientists can request dangerous biological agents from colleagues at other institutions poses a potential threat [9]. All laboratories, whether government, private, university, or hospital-associated, should be brought into line with strict regulations, particularly with handling sensitive biological agents [34]. However, the risk posed by black-market purchases or by the use of common household items still remains, especially with the risk of clandestine laboratories, such as the shed used by the Rajneeshees [14]. This poses a unique challenge to authorities attempting to prevent low-level bioterrorism attacks. However, a number of programs have begun to identify possible countermeasures and investigative leads that authorities may use against this threat. Vigilance, careful maintenance of stocks of risky agents, regular background checks, and a culture where employees are encouraged to raise their concerns are a key part of this antiterrorism strategy [34].

Many of the obstacles preventing a WMD-level bioterrorism attack are easily overcome when groups focus on smaller-scale objectives. Without the need to focus on long-term storage and advanced dissemination tactics, terrorists and bad actors require significantly less work to achieve “operational capability” [6]. *Salmonella *is uniquely positioned as a small-scale weapon of bioterrorism for bad actors [6], as demonstrated by the example of the Rajneeshees [14]. Dozens of outbreaks of *Salmonella *occur in the food supply each year; it is unlikely that the 1984 bioterrorism would have ever been discovered had investigators not stumbled across it a year later [6].

## Conclusions

The mass *Salmonella *poisoning by the Rajneeshees in 1984 remains the largest bioterrorism attack in the history of the United States. However, the cult serves as a poignant reminder of the damage that even small-scale acts of bioterrorism by VNSAs can inflict. Authorities and health officials should stay well aware of the threat that similar actors may pose to society as biotechnology improves and online spaces provide rapid dissemination of knowledge. Ultimately, the example of the Rajneeshees should serve as a warning for the future.
